# Disadvantages and benefits of evolved unicellularity versus multicellularity in budding yeast

**DOI:** 10.1002/ece3.5322

**Published:** 2019-07-09

**Authors:** Jennie J. Kuzdzal‐Fick, Lin Chen, Gábor Balázsi

**Affiliations:** ^1^ Department of Systems Biology The University of Texas MD Anderson Cancer Center Houston Texas; ^2^ Department of Biology and Biochemistry University of Houston Houston Texas; ^3^ Louis and Beatrice Laufer Center for Physical & Quantitative Biology Stony Brook University Stony Brook New York; ^4^ Department of Biomedical Engineering Stony Brook University Stony Brook New York

**Keywords:** budding yeast, clumping, evolutionary transition, experimental evolution, multicellularity, stress resistance

## Abstract

Multicellular organisms appeared on Earth through several independent major evolutionary transitions. Are such transitions reversible? Addressing this fundamental question entails understanding the benefits and costs of multicellularity versus unicellularity. For example, some wild yeast strains form multicellular clumps, which might be beneficial in stressful conditions, but this has been untested. Here, we show that unicellular yeast evolve from clump‐forming ancestors by propagating samples from suspension after larger clumps have settled. Unicellular yeast strains differed from their clumping ancestors mainly by mutations in the *AMN1* (Antagonist of Mitotic exit Network) gene. Ancestral yeast clumps were more resistant to freeze/thaw, hydrogen peroxide, and ethanol stressors than their unicellular counterparts, but they grew slower without stress. These findings suggest disadvantages and benefits to multicellularity and unicellularity that may have impacted the emergence of multicellular life forms.

## 1 INTRODUCTION

Multicellularity has evolved over 20 different times on Earth, leading to complex life forms in algae, animals, plants, and fungi (Grosberg & Strathmann, 2007). What forces contributed to the emergence and maintenance of multicellularity, and could reversals to unicellularity occur? Over 100 years ago, Louis Dollo hypothesized that phenotypic effects of evolutionary processes must be irreversible (Dollo, 1893), but this has since become controversial (Collin & Miglietta, 2008). Possible exceptions include the loss of flight in winged dinosaurs and birds (Paul, 2002), of parasitic traits in dust mites (Klimov & OConnor, 2013), or of protein sequence changes (McCandlish, Shah, & Plotkin, 2016; Soylemez & Kondrashov, 2012). How strictly Dollo's Law applies to major evolutionary transitions (Maynard Smith & Szathmáry, 1998), such as the relatively easy transition to multicellularity (Grosberg & Strathmann, 2007; Ratcliff, Denison, Borrello, & Travisano, 2012) is insufficiently explored, although a recent paper by Rebolleda‐Gomez and Travisano (2018) has begun to address this in the laboratory‐evolved snowflake yeast system. Addressing this problem requires charting the environment‐dependent downsides and benefits of unicellularity versus multicellularity. For example, cooperating “wrinkly spreader” *Pseudomonas fluorescens* bacteria form multicellular mats on the surface of liquids which improves access to oxygen, but noncontributing unicellular cheats regularly arise and cause colony collapse, subsequently acting as propagules (Hammerschmid, Rose, Kerr, & Rainey, 2014). More broadly, multicellularity may provide the benefits of dispersal in sparse nutrient conditions (Kuzdzal‐Fick, Foster, Queller, & Strassmann, 2007; Smith, Queller, & Strassmann, 2014), stress resistance (Smukalla et al., 2008), nutrient acquisition (Koschwanez, Foster, & Murray, 2011), and predator protection (Pentz, Limberg, Beermann, & Ratcliff, 2015). Implicitly, unicellularity is disadvantageous in such conditions—yet it improves growth without stress (Smukalla et al., 2008). We set out to study these trade‐offs in budding yeast.

Contrary to the classical definition of yeasts as single‐celled fungi, some *Saccharomyces cerevisiae* strains exhibit multicellular phenotypes (Andersen et al., 2014; Cap, Vachova, & Palkova, 2012; Reynolds & Fink, 2001), such as aggregation into flocs (Smukalla et al., 2008), flors (Zara, Zara, Pinna, Marceddu, & Budroni, 2009), and pattern formation on agar plates (Chen et al., 2014; Kuthan et al., 2003; Reynolds & Fink, 2001). Other yeast strains form multicellular “clumps” that differ from flocs and flors in their mechanism of formation and underlying genetics (Li et al., 2013). Instead of cell aggregation, clumps form by incomplete daughter cell separation, as budding continues while daughter cells remain attached to the mother cell (Kuranda & Robbins, 1991). Such yeast clumps aid nutrient acquisition in sucrose (Koschwanez et al., 2011), but their role in stress response is unclear.

Understanding the costs and benefits of social traits in yeast could elucidate general forces that maintain or convert unicellularity to multicellularity (Maynard Smith & Szathmáry, 1998) and back. The existence of unicellular and clumpy yeast in nature (Wloch‐Salamon, Plech, & Majewska, 2013) suggests condition‐dependent benefits and disadvantages, and bidirectional transitions between unicellularity and multicellularity. Could clumps provide protection from environmental stress as flocs do (Smukalla et al., 2008) while being disadvantageous in normal conditions? More broadly, could reverse transitions to unicellularity occur without cheaters, and what are the evolutionary forces that aid or prevent such reverse transitions?

To address these questions, here we compared how various environmental stressors affect the growth of genetically similar clump‐forming and unicellular “EvoTop” yeast cells that we obtained by reversing the strategy of “snowflake” yeast evolution (Ratcliff et al., 2012). Sequencing and comparing the genomes of the clumping ancestor and single‐celled “EvoTop” lines revealed unique missense and nonsense mutations in the *AMN1* gene, which is associated with multicellularity (Li et al., 2013; Yvert et al., 2003). Clump‐forming ancestral cell lines grew faster relative to untreated controls than EvoTop lines after exposure to rapid freeze/thaw, 1% ethanol, and 150 µM hydrogen peroxide stressors, indicating that clumping provides resistance to chemical and physical stresses. On the other hand, clumping hampered growth in the absence of stress, suggesting a trade‐off between the benefits and downsides of multicellularity versus unicellularity. Overall, this work sheds light on the genetic bases, as well as on the disadvantages and benefits of unicellularity versus clumping multicellularity in yeast, with implications for bidirectional transitions between other unicellular and multicellular life forms.

## 2 MATERIALS AND METHODS

### 2.1 Yeast strains

We used three strains of the budding yeast *S. cerevisiae* in this study. The first one was TBR1 (Σ1278b strain 10560‐23C; MATα, ura3‐52, his3::hisG, leu2::hisG), a segregant obtained by multiple crosses of baking strains “Yeast Foam” and 1422‐11D that carries 3.2 single‐nucleotide polymorphisms per kilobase compared to the standard laboratory strain S288c (Dowell et al., 2010). The second was the standard laboratory strain BY4742 (S288C‐derivative, MATα his3Δ1 leu2Δ0 lys2Δ0 ura3Δ0). The third one was KV38, a haploid strain obtained from the wild strain EM93, the ancestor of S288c, and source for 90% of its gene pool (Smukalla et al., 2008).

### 2.2 Fluorescent labeling of TBR1

The integration of GFP and mCherry reporters into TBR1 chromosomes was described previously (Chen et al., 2014). Briefly, *Escherichia coli* strains with either the pDN‐G1Gh (GFP) or the pDN‐G1Ch (mCherry) plasmids (both of which harbor the ampicillin resistance selection marker) were incubated in LB media with ampicillin (1:1,000) at 37°C for 6–8 hr. The plasmids were then extracted by midi prep (QIAGEN), linearized, and purified. They were then transformed into the native *GAL1* locus of the TBR1 strain, using the histidine auxotrophic marker. Transformation was performed using a modified lithium acetate procedure as described before (Chen et al., 2014). Synthetic drop‐out (SD‐his‐tryp) plates were then used for selection (all reagents from Sigma, Inc.). Once established, TBR1‐GFP and TBR1‐mCherry strains were incubated in SD‐his‐tryp + 2% galactose at 30°C, shaking at 300 rpm (LabNet 311DS shaking incubator). The TBR1‐GFP and TBR1‐mCherry strains were imaged using a Nikon TE2000 fluorescence microscope. Cells were counted with a Becton‐Dickinson FACScan flow cytometer during the experiment and manually from microscope images afterward.

### 2.3 Selection for unicellular yeast

To initiate three replicate lines of the haploid yeast strain TBR1, we inoculated three tubes of 2 ml yeast peptone dextrose (YPD) medium (10 g yeast extract, 20 g bacto peptone, 2% glucose per L) with single TBR1 colonies. We allowed each culture to grow overnight, froze aliquots of these “ancestral” cultures (TBR1 A, B, and C), and then prepared two 100× dilutions of each culture in 2 ml YPD to obtain matched pairs of TBR1 A, B, and C for starting the EvoTop and EvoControl treatment lines. Each line reached stationary phase by growing for 24 hr in a shaking LabNet 311DS incubator at 300 rpm at 30°C. After removal from the shaking incubator, large clumps should settle faster than single cells and small clumps. For the EvoTop treatment lines, we vortexed each tube before allowing them to remain in a 30°C MyTemp Mini Digital static incubator for 45 min before taking a 20 µl sample from the top of the liquid culture to inoculate a new tube with 2 ml of YPD for growth overnight under the previously described conditions. We performed the selection procedure each day over 4 weeks, for a total of 28 rounds of selection and resuspension. Over the course of the selection experiment, we maintained parallel EvoControl lines by vortexing each strain before selecting 20 µl samples for each transfer into new tubes with 2 ml YPD. We froze ancestral samples at the beginning of the experiment and samples of each EvoTop and EvoControl line (700 μl cell solution with 300 μl 80% glycerol) in a −80°C freezer every 3–4 days, including the final cultures. We followed the same protocol with another haploid clump‐forming strain, KV38 (Smukalla et al., 2008), and with the haploid unicellular laboratory strain BY4742, an S288c derivative (Brachmann et al., 1998), as a control.

### 2.4 Estimating cell and clump sizes

To determine cell size and clump size of each EvoTop, EvoControl, and ancestral line, we used a Nexcelom Cellometer Auto M10 automated cell counter to analyze 10× dilutions in YPD of overnight cultures (grown in YPD solution in a 300 rpm shaking LabNet 311DS incubator at 30°C) started from frozen samples of each line, from the ancestral state through to the final frozen sample. Diameters from 10× dilutions of “live” samples were also measured a number of times toward the beginning and end of the selection experiment. By default, the Nexcelom Cellometer software declusters clumps and measures and counts individual cells within them. Obviously, clumps are larger than cells, so the cell counting parameters must have smaller maximum diameters than the clump counting parameters. It is also very important to note that clumps were measured with the “Do not Decluster Clumps” parameter selected, which counts and measures each clump as a whole unit. Specifically, for cell size, we set the Cellometer Auto cell type parameters as follows: cell diameter minimum of 2.0 μm and maximum of 9.0 μm, roundness of 0.10, and contrast enhancement of 0.40, with a decluster edge factor of 0.5 and Th factor of 1.0. We measured clumps with the following parameters: cell diameter minimum of 2.0 μm and maximum of 40.0 μm, roundness of 0.10, and contrast enhancement of 0.40, with “Do not Decluster Clumps” selected. We increased the maximum cell diameter of clumps to 100 μm for samples where the program indicated that some clumps were larger than 40 μm in diameter.

We combined clump and cell diameter “live” data gathered during the experiment with data from the lines taken out of the freezer and then averaged together all clump or cell data points by line (within strain and treatment) for days that had both, including the initial “ancestor” and final “EvoTop” and “EvoControl” days. We used the *userfriendlyscience* version 0.7.2 package in R version 3.6.0 (Peters, 2018; R Core Team, 2018) to perform one‐way ANOVAs analyzing the effect of treatment on clump diameter or the effect of treatment on cell diameter for TBR1, BY4742, and KV38, followed by Games–Howell post hoc tests.

### 2.5 Estimating cell and clump sizes of KV38

Flocculation is another form of multicellularity that could potentially confuse the results. It is well known that yeast flocs, but not clumps, can be separated by ethylenediaminetetraacetic acid (EDTA). When we started the selection experiment, the KV38 ancestor did not appear to flocculate, so EDTA was not added to the sample we obtained live ancestral clump and cell size data from. However, KV38's tendency to flocculate fluctuated throughout the selection experiment, so we added 5 mM EDTA to break up flocs before measuring clump and cell size. We also imaged the cultures started from frozen samples of each line in YPD with 5 mM EDTA.

### 2.6 TBR1 sequencing analysis

The DNA from the ancestor and each of the three TBR1 EvoTop stress tested isolates was extracted from cultures started from individual colonies that were anticipated to be clonal. Clonal mutations are expected to be present at a minimum of 80%–90% in clonal population samples analyzed as if they were polymorphic (Saxer et al., 2014). Since the TBR1 A EvoTop isolate appeared to be founded by two clones, with the lower frequency clone present at about 18%, we reasoned that this clone could have other clonal mutations present at as low as 14% (=0.8 × 18%; Table 1).

**Table 1:** Unique mutations in the stress tested and sequenced TBR1 EvoTop isolates. Mutations shown in bold were also called in the EvoTop line the isolate came from.

EvoTop isolate	Seq id	Position	Mutation	Annotation	Gene	Description	Breseq freq	LoFreq* freq
TBR1 A	gi295413810gbACVY01000005.1	346,739	C→T	G439S (GGC→AGC)	*YBR068C_CDS_BAP2 ←*	High‑affinity leucine permease; functions as a branched‑chain amino acid permease involved in the uptake of leucine, isoleucine, and valine; contains 12 predicted transmembrane domains	24.13%	23.70%
	**gi295413810gbACVY01000005.1**	**527,059**	**T→G**	M1R (ATG→AGG)	*YBR158W_CDS_AMN1 →*	Protein required for daughter cell separation, multiple mitotic checkpoints, and chromosome stability; contains 12 degenerate leucine‑rich repeat motifs; expression is induced by the Mitotic Exit Network (MEN)	72.59%	72.11%
	**gi295413810gbACVY01000005.1**	**527,116**	**C→A**	S20* (TCA→TAA)	*YBR158W_CDS_AMN1 →*	Protein required for daughter cell separation, multiple mitotic checkpoints, and chromosome stability; contains 12 degenerate leucine‑rich repeat motifs; expression is induced by the Mitotic Exit Network (MEN)	18.93%	18.13%
	**gi295413787gbACVY01000028.1**	**56,138**	**G→C**	S174C (TCC→TGC)	*YER088C_CDS_DOT6 ←*	Protein involved in rRNA and ribosome biogenesis; binds polymerase A and C motif; subunit of the RPD3L histone deacetylase complex; similar to Tod6p; has chromatin specific SANT domain; involved in telomeric gene silencing and filamentation	23.60%	22.89%
	gi295413784gbACVY01000031.1	47,990	C→T	E1693K (GAG→AAG)	*YLL040C_CDS_VPS13 ←*	Protein of unknown function; heterooligomeric or homooligomeric complex; peripherally associated with membranes; homologous to human COH1; involved in sporulation, vacuolar protein sorting, and protein‑Golgi retention	64.11%	61.46%
TBR1 B	**gi295413810gbACVY01000005.1**	**528,271**	**C→G**	T405R (ACG→AGG)	*YBR158W_CDS_AMN1 →*	Protein required for daughter cell separation, multiple mitotic checkpoints, and chromosome stability; contains 12 degenerate leucine‑rich repeat motifs; expression is induced by the Mitotic Exit Network (MEN)	100.00%	97.86%
	gi295413808gbACVY01000007.1	431,329	C→A	Q1442K (CAA→AAA)	*YJL005W_CDS_CYR1 →*	Adenylate cyclase; required for cAMP production and cAMP‑dependent protein kinase signaling; the cAMP pathway controls a variety of cellular processes, including metabolism, cell cycle, stress response, stationary phase, and sporulation	100.00%	96.89%
	gi295413805gbACVY01000010.1	84,670	C→A	P71T (CCA→ACA)	*YOR255W_CDS_OSW1 →*	Protein involved in sporulation; required for the construction of the outer spore wall layers; required for proper localization of Spo14p	100.00%	98.95%
	gi295413792gbACVY01000023.1	63,441	T→C	Intergenic (‑405/‑334)	*YIL023C_CDS_YKE4 ←/→ YIL022W_CDS_TIM44*	Zinc transporter; localizes to the ER; null mutant is sensitive to calcofluor white; leads to zinc accumulation in cytosol; ortholog of the mouse KE4 and member of the ZIP (ZRT, IRT‑like Protein) family/Essential component of the Translocase of the Inner Mitochondrial membrane (TIM23 complex); tethers the import motor and regulatory factors (PAM complex) to the translocation channel (Tim23p‑Tim17p core complex)	100.00%	96.43%
	gi295413769gbACVY01000046.1	1,436	G→T	Intergenic (−/−)	−/−	−/−	100.00%	100.00%
TBR1 C	**gi295413810gbACVY01000005.1**	**528,545**	**A→C**	K496N (AAA→AAC)	*YBR158W_CDS_AMN1 →*	Protein required for daughter cell separation, multiple mitotic checkpoints, and chromosome stability; contains 12 degenerate leucine‑rich repeat motifs; expression is induced by the Mitotic Exit Network (MEN)	100.00%	96.57%

We used high‐throughput whole‐genome sequencing to identify the mutations underlying the change from clumping to “unicellular” phenotypes in our TBR1 EvoTop lines and isolates. We followed QIAGEN's “Purification of total DNA from yeast using the DNeasy Blood & Tissue Kit” protocol to obtain high‐quality genomic DNA from the TBR1 ancestor, EvoControl, and EvoTop lines and isolates. Columbia Genome Center used these samples to run whole‐genome sequencing on an Illumina HiSeq 2000 v3 instrument, and they aligned the obtained reads in fastq files to the Σ1278b reference genome (Dowell et al., 2010) using BWA‐mem.

We prepared the provided aligned bam files for analysis by adapting the Genome Analysis Toolkit's (GATK) best practices procedures (Van der Auwera et al., 2013): mark duplicates with REMOVE_DUPLICATES = true (Picard‐tools 1.119), indel realignment (GATK 3.3‐0), and base quality recalibration (GATK 3.3‐0). Then, we added alignment qualities with lofreq_star‐2.1.2 (Wilm et al., 2012). Because the EvoControl and EvoTop populations were heterogeneous, we looked for low frequency mutations in the genomic DNA of all 10 samples (TBR1 ancestor, TBR1 A, B, and C EvoControl, EvoTop, and EvoTop isolates) with lofreq_star‐2.1. In addition, we used the breseq‐0.26.1 pipeline (Deatherage & Barrick, 2014) with bowtie2‐2.2.6 and the –j2, ‐p, ‐c options to align fastq files to the Σ1278b reference genome (Dowell et al., 2010) in order to independently identify variants in each of the 10 lines. To identify mutations in the EvoTop isolates that were not called in the ancestor, we used the vcf‐isec command of VCFtools 0.1.12b (Danecek et al., 2011) with option –c for LoFreq* files and gdtools SUBTRACT for breseq files. We used vcf‐isec –f –n+2 to identify which of these mutations were called by both LoFreq* and breseq and then ran LoFreq*'s uniq command to identify which of these mutations were really unique from the ancestor. To help determine the potential impact of these mutations, we used gdtools ANNOTATE and the Sigma1278b_ACVY01000000.gff file deposited at yeastgenome.org by Dowell and colleagues (2010). We analyzed all 10 lines as polymorphic populations so that we could directly compare the results. Table 1 (adapted from breseq's “Predicted mutations” tables) lists the mutations in the TBR1 EvoTop isolates that were unique from the ancestor and were called by both LoFreq* and breseq with a minimum frequency of 14% (see above).

### 2.7 Assays of stress resistance

To compare the differences in stress response between the clump‐forming TBR1 ancestor and its “single‐celled” descendants, we isolated colonies from each ancestral and EvoTop TBR1 line (A, B, and C) that were good representatives of these phenotypes (see “TBR1 isolates” section below and Figure 5). We diluted exponentially growing cells started from individual colonies and placed 1.5 ml of each dilution into two separate 2 ml microcentrifuge tubes to start a control and experimental stress treatment as described below. We used R version 3.6.0 and R package emmeans version 1.3.4 to run three‐way full‐factorial ANOVAs and Tukey post hoc tests to analyze variant, line, and stress effects on the relative growth of TBR1 and BY4742 cells and contrast estimated marginal means.

### 2.8 TBR1 isolates

For each TBR1 ancestor and EvoTop variant and line (A, B, and C), we took six colonies and used each to inoculate 1 ml SD‐tryp + 2% glucose (6.7 g nitrogen base, 1.92 g drop‐out supplements without tryp, 2% glucose per L) tubes for overnight growth in a shaking 30°C 300 rpm incubator. We froze isolates (700 μl SD with 300 μl 80% glycerol) and prepared dilutions in SD‐tryp + 2% glucose for exponential growth overnight. The following day, we used the Nexcelom Cellometer Vision CBA “Clumpy Yeast” parameters to measure clump diameters, distributions, and concentrations of isolates. Isolates with extremely low concentrations (<1 × 10^6^ clumps/ml) were not considered for further use. We took average clump diameter and clump size distribution into account to determine which ancestor and EvoTop isolates to use in our stress tests. We chose EvoTop isolates with small clumps that lacked larger clumps in their distribution data. For ancestor isolates, we chose ones with large clumps and distributions that lacked very small clumps. Isolates shown in purple (Figure 5) were used for stress tests. TBR1 C ancestor isolate 2 (green) was used in the first freeze/thaw experiment, but TBR1 C ancestor isolate 5 (purple) was used in all other stress test replicates.

#### 2.8.1 Testing for stress resistance: Freeze/thaw

For the freeze/thaw treatment, we rapidly froze cells in a dry ice and ethanol bath for 5 min and immediately thawed them in room temperature water. During this time, the remaining 1.5 ml cell samples stayed on the bench top as controls. We prepared 3× dilutions of each culture by adding 1 ml of cells to 2 ml SD‐tryp + 2% glucose in 5 ml polystyrene round‐bottom yeast culture tubes and transferred 1 ml of each sample into new yeast culture tubes. We placed the 2 ml samples back into a 300 rpm Labnet 311DS shaking incubator at 30°C, and prepared Nexcelom automated cell counter slides from each ancestral and EvoTop cell line from the 1 ml aliquots. In order to break up clumps and obtain accurate cell counts, we used a Qsonica Q55 sonicator to administer 20 pulses of 30% amplitude sonication to each of the 1 ml aliquots, which were placed on ice between each set of 10 pulses. We subsequently acquired clump diameter data before and after sonication, along with cell concentration data with a Nexcelom Cellometer Vision automated cell counter using the same parameters as described above for the Cellometer Auto (with the additional background adjustment parameter for cell measurements set at 1.0). After 1.5 hr of growth in the incubator, we prepared new 1 ml aliquots of each culture and followed the previously described protocol to obtain clump diameter and cell concentration data. We then calculated the proportion change in cell concentration for the EvoTop single cells and ancestral clumps under freeze/thaw and control conditions as follows:

(final concentration − initial concentration)/initial concentration.

To obtain the relative growth of stressed cells compared to their controls, we divided the proportion change of each freeze/thaw sample by the proportion change in its control counterpart:

(proportion change freeze/thaw)/(proportion change control).

We followed the same protocol to examine the effects of freeze/thaw stress on our BY4742 A, B, and C lines, but determined that sonication was not needed to obtain countable cells.

#### 2.8.2 Testing for stress resistance: 150 µM hydrogen peroxide and 1% exogenous ethanol

In order to determine whether clumps of yeast are more resistant than single cells to the chemical stressors hydrogen peroxide (H_2_O_2_) and ethanol, we followed a similar protocol to the one described for freeze/thaw. We diluted exponentially growing cells started from individual colonies of TBR1 A, B, and C EvoTop and ancestor isolates to 3.5 × 10^6^ cells/ml in SD‐tryp + 2% glucose. We made 2× dilutions of the cells for control and 150 µM H_2_O_2_ or 1% ethanol treatments by adding 1 ml of cells to prepared 5 ml yeast culture tubes containing 1 ml SD‐tryp + 2% glucose for controls and either 300 µM H_2_O_2_ in 1 ml SD‐tryp + 2% glucose or 2% ethanol in 1 ml SD‐tryp + 2% glucose for stress treatments. To obtain aliquots for initial counts, we transferred 1 ml of each culture into new yeast culture tubes. Then, we placed the remaining samples back into the 300 rpm shaking LabNet 311DS incubator at 30°C. We allowed the single‐cell and clump lines to grow for 1.5 hr in a 300 rpm LabNet 311DS shaking incubator at 30°C. To calculate the proportion change in concentration of EvoTop single cells and ancestral clumps, we used sonicated cell counts obtained from a Nexcelom Cellometer Vision automated cell counter in the manner previously described for the freeze/thaw experiment. We also calculated and analyzed the relative growth of stressed cells compared to their unstressed controls for hydrogen peroxide or ethanol samples as previously described for the freeze/thaw experiment. We followed the same protocols with our BY4742 A, B, and C EvoTop and ancestral lines, but did not sonicate the cells for counting.

### 2.9 TBR1 and BY4742 unstressed growth rates

We ran a three‐way ANOVA (lm) in R version 3.6.0 to analyze the effects of variant, phenotype, and line, and variant by phenotype, and phenotype by line interactions on the growth (proportion change over an hour and a half) of unstressed TBR1 and BY4742 clumps and single cells. As we will discuss in the Results section, TBR1 ancestral clusters were significantly larger than TBR1 EvoTop clusters, while BY4742 ancestral multiplets were smaller than BY4742 EvoTop multiplets. Therefore, for this analysis, we considered TBR1 ancestors and BY4742 EvoTops to be “clump‐forming”, and we considered TBR1 EvoTops and BY4742 ancestors to be “single‐celled”.

## 3 RESULTS

### 3.1 The TBR1 yeast strain shows the clumping phenotype

To explore the bidirectional transitions and fitness effects of unicellularity versus multicellularity in yeast, we focused on the haploid *S. cerevisiae* strain TBR1 (see the Materials and Methods section), a segregant obtained by multiple crosses of baking strains that carries thousands of polymorphisms relative to the standard laboratory strain S288c (Dowell et al., 2010) and develops wrinkly patterns on soft agar plates (Chen et al., 2014; Reynolds & Fink, 2001). We asked whether TBR1 cells would also be capable of clump formation by incomplete separation. Thus, we compared phenotypes related to clump formation in the TBR1 strain and the standard laboratory strain BY4742, a haploid‐derivative of S288c.

**Figure 1:**
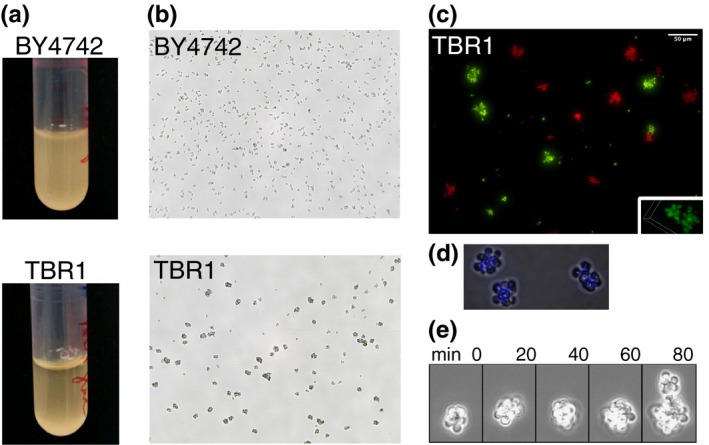
TBR1 cells form clumps by incomplete separation. (a) Settling patterns, 45 min after shaking. Yeast cells were kept in YPD and shaken overnight at 300 rpm at 30°C, moved to a static incubator for 45 min, and imaged. (b) Nonclumping laboratory strain BY4742 (top) and clumpy TBR1 strain (bottom) imaged on a Nexcelom Cellometer M10 at 10× magnification. (c) Lack of aggregation in TBR1. Two samples of the same TBR1 strain were tagged with the yEGFP and mCherry reporters expressed from the *GAL1* promoter. An initially 1:1 mixture was resuspended daily in appropriate SD medium for auxotroph selection + galactose. After 7 days, clumps were either purely red (*n* = 205) or green (*n* = 129), indicating lack of aggregation (Nikon TE2000, 20×). Isolated single cells (single or in doublets) were also present (*n* = 414 red; *n* = 461 green). Some other single cells overlapped with clumps (*n* = 35 green cells on red clumps; *n* = 14 red cells on green clumps). Small panel: confocal image, 60× magnification. (d) Calcofluor white stained bud necks in TBR1 indicate that clumps form from incomplete daughter cell separation, as opposed to aggregation. (e) Clump division: A TBR1 clump divides into two new clumps in static medium, without shaking. Images were taken 20 min apart in a Nikon Biostation CT, at 40× magnification. The TBR1 clump grows over 60 min as new daughter cells remain attached. At 80 min, a small clump (on top) breaks off from the larger parent clump

Strains capable of clump formation tend to settle to the bottom of the culture tube over time. Therefore, we first visually tested the settling of these two yeast strains 45 min after removal from the shaking incubator. We noticed that the entire liquid culture medium was still uniformly turbid for the laboratory strain BY4742. In contrast, the TBR1 strain formed a vertical gradient of turbidity, increasing from top to bottom (Figure 1a). Next, we examined by microscopy how these different settling behaviors correlated with sizes and shapes at the cellular level. The standard laboratory strain BY4742 appeared mainly as single cells, doublets, and occasional triplets (Figure 1b, top), whereas the TBR1 strain appeared as small, but compact and regular‐shaped clumps and multiplets (Figure 1b, bottom), suggesting that clumps must split or must shed cells to limit their sizes.

Besides clump formation by incomplete separation (Figure 1d,e), aggregation (flocculation) balanced by splitting/shedding is another mechanism that could give rise to the multicellular structures seen in Figure 1b. If this were the case, then cells from the same strain with two different labels should mix within multicellular structures over time as the structures stick together (Smukalla et al., 2008). To test this, we labeled two samples of the same TBR1 clumping strain with chromosomally integrated red and green fluorescent reporters, and followed approximately equal numbers of red and green cells over time by fluorescence microscopy. We observed no color‐mixing within multicellular structures even after 7 days of co‐culture (Figure 1c), indicating that TBR1 cells grow as clumps by incomplete separation, and do not flocculate by aggregation. Additionally, we reasoned that bud necks located inside the multicellular structures would support incomplete separation over aggregation. To test this idea, we used calcofluor white to stain bud necks within the multicellular structures. Indeed, we observed bud necks only inside multicellular structures, providing further support for clump formation by incomplete separation (Figure 1d).

Diploid multicellular “snowflake” yeast were found to divide as multicellular units, splitting into two smaller “snowflakes” (Ratcliff et al., 2012). To see whether this applied to TBR1 clumps, we tested how clumps multiply without shaking, to avoid the shedding of single cells due to physical shear. The TBR1 strain existed nearly exclusively in the form of clumps and grew by clump (rather than single‐cell) division in these conditions (Figure 1e). This further supported the notion that the TBR1 haploid strain predominantly exists in the form of multicellular clumps that originate from incomplete daughter cell separation and divide as units.

### 3.2 Multicellular to unicellular transition by laboratory evolution

Next, we asked if TBR1 clumps can undergo a reverse evolutionary transition to unicellularity. Such a transition should result in nonclumping cells that are genetically as similar as possible to their clumping ancestors. These evolved cells would also facilitate testing the benefits and potential costs of clumping during environmental stress: being genetically similar to the ancestors should minimize (although not eliminate) contributions to stress resistance from mechanisms and genes unrelated to clumping.

**Figure 2:**
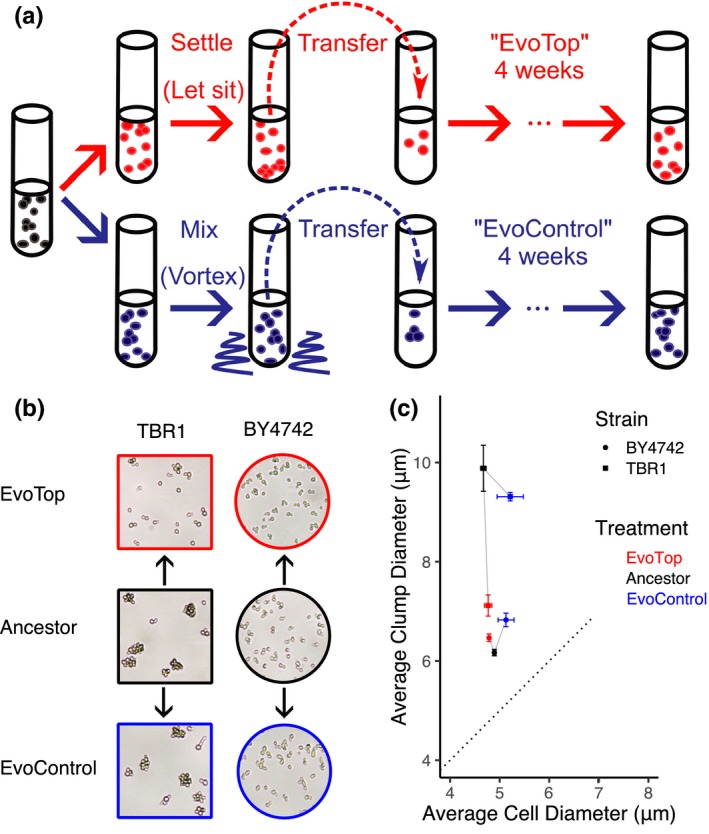
Experimental evolution of unicellularity. (a) Experimental evolution procedure repeated daily over 4 weeks, after ancestral cells were split into “EvoTop” and “EvoControl” cells on the first day. “EvoTop” cells were vortexed and then allowed to settle for 45 min before resuspending a small sample from the top of the liquid culture. “EvoControl” cells were vortexed before resuspending the same way. (b) Representative images of TBR1 and BY4742 EvoTop and EvoControl cells compared to their ancestors. The shapes and colors of image frames correspond to the shapes and colors in panel (c). (c) Clump size (diameter) of TBR1 EvoTop (red square) decreased compared to TBR1 ancestor (black square) and TBR1 EvoControl (blue square) variants: one‐way ANOVA, *F*(2, 6) = 23.91, *p* = 0.001; Games–Howell post hoc test, *p* < 0.05. In contrast, BY4742 EvoTop cluster diameter (red circle) was larger than BY4742 ancestor (black circle), but this was not significant at a 0.05 alpha level: one‐way ANOVA, *F*(2, 6) = 11.53, *p* = 0.009; Games–Howell, *p* = 0.088, error bars indicate *SEM*. BY4742 EvoControl clusters (blue circle) were significantly larger than BY4742 ancestral clusters (black circle): Games–Howell, *p* < 0.05. The average cell diameter did not vary significantly with selection treatment within TBR1 (one‐way ANOVA *F*(2, 6) = 3.18, *p* = 0.114) or within BY4742 (one‐way ANOVA *F*(2, 6) = 3.40, *p* = 0.103). The dotted line indicates a 1:1 cell diameter to clump diameter ratio, where nonbudding unicellular strains would theoretically lie

**Figure 3:**
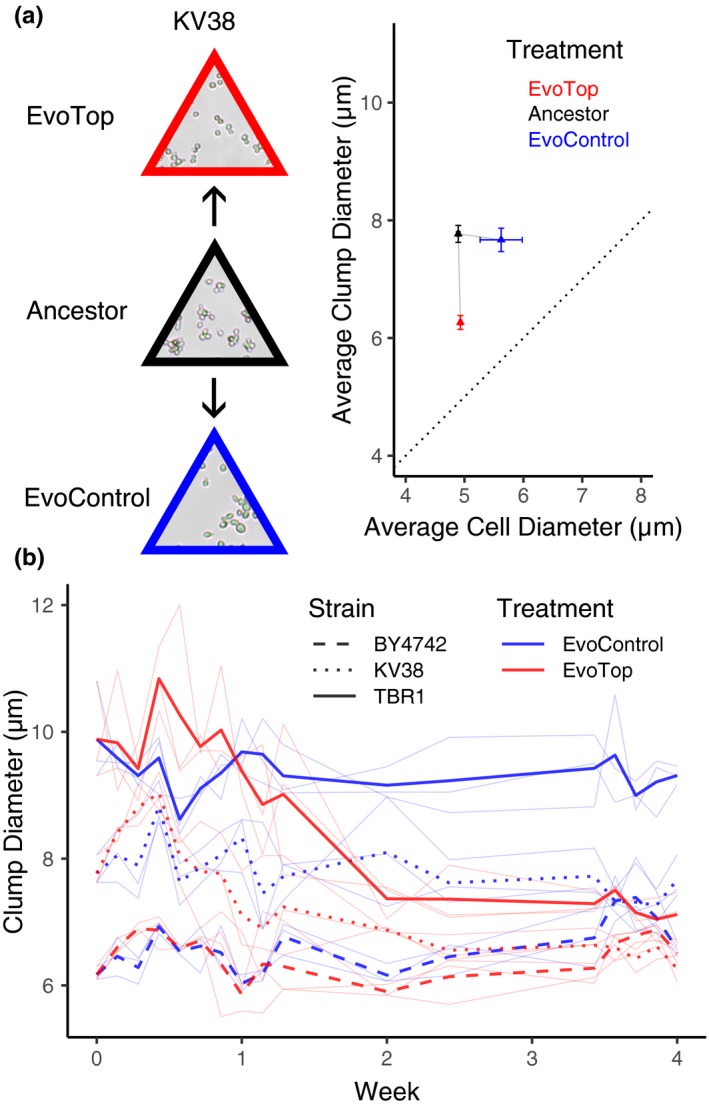
EvoTops derived from clumping strains are smaller than their ancestors. (a) KV38's mean EvoTop (red triangle) clump diameter was significantly smaller than that of the ancestral (black triangle) and EvoControl lines (blue triangle; one‐way ANOVA *F*(2, 6) = 28.61, *p* = 0.001; Games–Howell post hoc test, *p* < 0.05, error bars indicate *SEM*), which did not vary significantly from each other (*p* > 0.05). The diameters of KV38's EvoControl cells, ancestral cells, and EvoTop cells did not vary significantly from each other (one‐way ANOVA *F*(2, 6) = 3.93, *p* = 0.081). The dotted line indicates a 1:1 cell diameter to clump diameter ratio, where nonbudding unicellular strains would theoretically lie. (b) TBR1 (solid bold lines) and KV38 (dotted lines) EvoTop clumps (red lines, week 4) were significantly smaller than their corresponding ancestral (week 0) and EvoControl clumps (blue lines, week 4), while the average BY4742 (dashed lines) EvoControl and EvoTop clump diameters (week 4) tended to be larger than their ancestor's (week 0). Replicates are shown in thin light blue and red lines

To induce a reverse transition and obtain two yeast strains that differ in their clumping phenotype but are otherwise genetically similar, we derived a nonclumping, unicellular variant from the “ancestral” TBR1 strain by laboratory evolution. In 2012, Ratcliff and colleagues selected for multicellular diploid “snowflake” yeast by continuously propagating the yeast that settled most rapidly to the bottom of their cultures (Ratcliff et al., 2012). Here, we reversed that selection process by continuously selecting for single cells or smaller clumps from the tops of our cultures, which remained suspended after the larger clumps settled over 45 min (Figure 2a). We refer to these evolving cell lines as the “EvoTop” variant. We also propagated in parallel an “EvoControl” variant, with cell lines that had the same TBR1 ancestor, but were mixed thoroughly by vortexing before every resuspension. To assess the effect of the counter‐gravitational selection for unicellularity, we tracked the average size (diameter) of uni‐ and multicellular structures. A one‐way ANOVA revealed a significant effect of selection treatment (*F*(2, 6) = 23.91, *p* = 0.001) on TBR1 clump diameters. A Games–Howell post hoc test indicated that after 4 weeks of daily selection, the TBR1 EvoTop variant formed significantly smaller clumps (*M* = 7.118 µm, *SD* = 0.369) than the ancestral (*M* = 9.884 µm, *SD* = 0.802; *p* = 0.029) or randomly chosen EvoControl (*M* = 9.311 µm, *SD* = 0.148; *p* = 0.008) variants (Figures 2 and 3b), suggesting a reverse evolutionary transition toward unicellularity under the counter‐gravitational selection. EvoControl clump size did not differ significantly from ancestral clumps (Games–Howell, *p* = 0.547).

**Figure 4:**
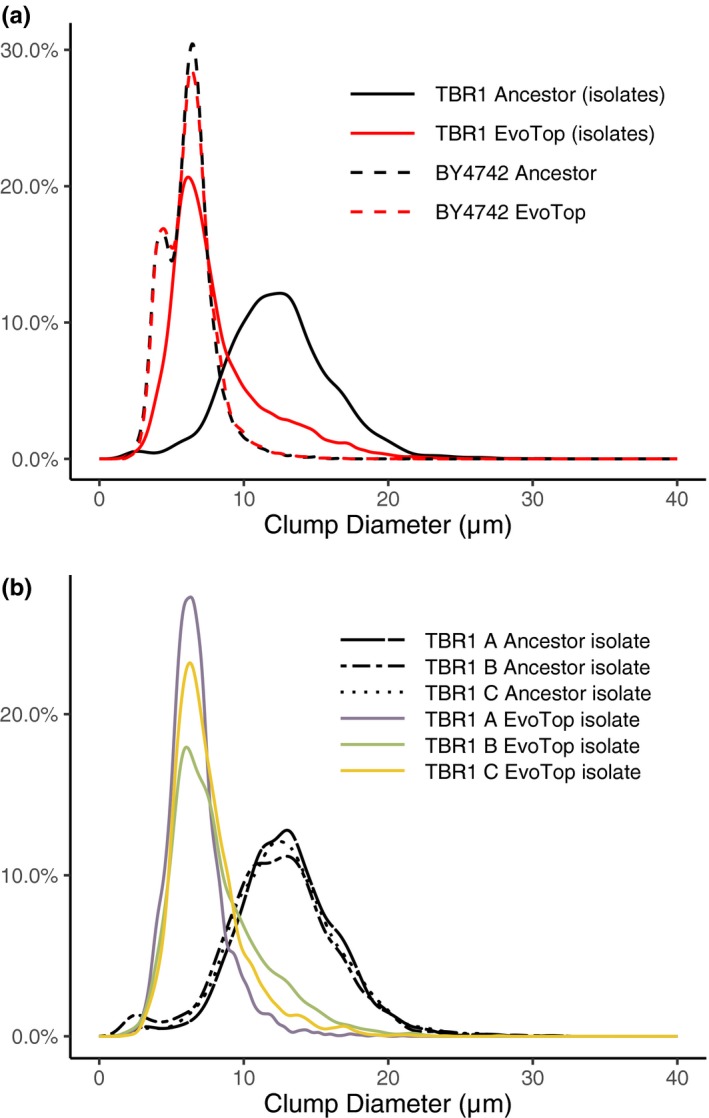
TBR1 EvoTop diameter distributions shifted left, while BY4742 remained similar. (a) In SD‐tryp + 2% glucose medium, the clump diameter distribution of TBR1 EvoTop stress tested isolates (solid red line) approached that of the unicellular laboratory strain BY4742 (dashed lines). Clump distributions are from the combined data points of all lines (A, B, and C) within a variant (TBR1 ancestor, TBR1 EvoTop, BY4742 ancestor, and BY4742 EvoTop). (b) TBR1 EvoTop stress tested isolates have similar clump size distributions. In SD‐tryp + 2% glucose medium, the TBR1 B EvoTop isolate (green line) and the TBR1 C EvoTop isolate (orange line) have clump diameter distributions that are similar to that of the TBR1 A EvoTop isolate (purple line)

There are at least two possible ways for clump size to decrease during evolution: clumps could contain fewer or smaller cells. To distinguish between these possibilities, we compared the cellular diameters of TBR1 ancestral, EvoControl, and EvoTop variants. As described under “Estimating cell and clump sizes” in the Materials and Methods section, the Cellometer software can decluster clumps to identify individual cells. A one‐way ANOVA found no significant effect of selection treatment (*F*(2, 6) = 3.18, *p* = 0.114) on the cell diameters of the EvoControl (*M* = 5.213 µm, *SD* = 0.458), ancestral (*M* = 4.672 µm, *SD* = 0.098), and EvoTop (*M* = 4.768 µm, *SD* = 0.129) variants (Figure 2c). Estimating the average number of cells per clump by dividing the average volume of “spherical” clumps by the average volume of “spherical” cells indicates that the decrease in the TBR1 EvoTop clump sizes was driven by an ~2.8 fold decrease in average cell number from 9.47 to 3.33 cells per clump at full packing density (=1), rather than smaller cell size within clumps. This also holds true for TBR1 EvoControl clumps, where the average number of cells per EvoControl clump was ~1.7 times smaller than that of the ancestor, at 5.70 cells per clump versus 9.47. As a comparison, the “unicellular” laboratory strain BY4742 averages 2 cells per “clump” following these calculations, although all populations contain a range of clump sizes (Figure 4a). Isolates from the TBR1 EvoTop lines exhibited phenotypes approaching that of the BY4742 unicellular laboratory strain (Figures 2b,c and 4); therefore, we refer to them as “unicellular” EvoTop TBR1 cells. To test the robustness of these findings, we repeated the evolution experiment for KV38, another clump‐forming strain (Smukalla et al., 2008).

Similar to TBR1, the number of cells per clump decreased for the KV38 EvoTop variant, confirming that our selection procedure could cause reverse transitions to unicellularity in different yeast cell lines (Figure 3). A one‐way ANOVA revealed a significant effect of selection treatment (*F*(2, 6) = 28.61, *p* = 0.001) on KV38 clump size. A Games–Howell post hoc test indicated mean EvoTop clump diameter (*M* = 6.263 µm, *SD* = 0.205) was significantly smaller than that of the ancestral (*M* = 7.770 µm, *SD* = 0.249; *p* = 0.003) and EvoControl (*M* = 7.667 µm, *SD* = 0.345; *p* = 0.015) variants, which did not vary significantly from each other (*p* = 0.911; Figure 3). A one‐way ANOVA did not show a significant effect of selection treatment (*F*(2, 6) = 3.93, *p* = 0.081) on KV38 cell size, indicating that the diameters of EvoControl cells (*M* = 5.622 µm, *SD* = 0.619), ancestral cells (*M* = 4.894 µm, *SD* = 0.049), and EvoTop cells (*M* = 4.930 µm, *SD* = 0.037) were not significantly different from each other. Assuming a packing density of 1, the average ancestral KV38 clump contained nearly twice (~1.95) as many cells per clump (4.00) as the KV38 EvoTop variant (2.05) and ~1.57 times as many cells per clump as the EvoControl variant (2.54). Therefore, these results indicate that not only were KV38 EvoTop clumps comprised of fewer cells than KV38 ancestral clumps, so were KV38 EvoControl clumps. Along with the TBR1 results, this suggests that having fewer cells per clump might be beneficial under the growth conditions of our experiment, with or even without settling‐based selection.

Finally, as a control, we also conducted an identical selection experiment on the standard laboratory strain BY4742. As opposed to TBR1 and KV38, the ancestral cultures of this unicellular strain contained mainly single cells, intermixed with occasional multiplet structures of two or three cells (doublets or triplets). As expected, the mean “multiplet diameter” of the EvoTop lines did not decrease over time (Figure 3b). Surprisingly, however, the presence of small multiplets (mainly doublets and triplets) increased over time in the BY4742 EvoControl and EvoTop variants. A one‐way ANOVA indicated a significant effect of selection treatment (*F*(2, 6) = 11.53, *p* = 0.009) on clump (multiplet) diameters. A Games–Howell post hoc test revealed that BY4742 EvoControl multiplets (*M* = 6.828 µm, *SD* = 0.234) were significantly larger than BY4742 ancestral multiplets (*M* = 6.169 µm, *SD* = 0.112, *p* = 0.047; Figure 2c), but did not differ significantly from EvoTop multiplets (*p* = 0.189). Like BY4742 EvoControl multiplets, BY4742 EvoTop multiplets (*M* = 6.468 µm, *SD* = 0.133) tended to be larger than BY4742 ancestral multiplets, but this difference was not significant at a 0.05 alpha level (Games–Howell, *p* = 0.088). A one‐way ANOVA indicated that the effect of selection treatment (*F*(2, 6) = 3.40, *p* = 0.103) on mean BY4742 cell diameter was not significant among EvoControl cells (*M* = 5.128 µm, *SD* = 0.278), ancestral cells (*M* = 4.893 µm, *SD* = 0.058), and EvoTop cells (*M* = 4.787 µm, *SD* = 0.017; Figure 2b,c), so the increase in BY4742 EvoTop and EvoControl “multiplet size” was not driven by an increase in cell size. This trend may suggest some competitive advantage of such multiplets over single cells under these conditions, such as covering bud scars, which might cause vulnerability during vortexing (Chaudhari, Stenson, Overton, & Thomas, 2012).

In summary, by utilizing the settling rate differences among clumps of varying size within a population, we were able to select for unicellular yeast from clump‐forming haploid ancestors. Surprisingly, even in the absence of settling‐based selection, clump sizes decreased slightly. In addition, we observed a mild tendency toward multiplet formation for the initially unicellular BY4742 laboratory strain.

### 3.3 Genetic bases of unicellularity

To determine the genetic changes underlying the multicellular‐to‐unicellular transition, we performed whole‐genome sequencing on the TBR1 ancestor and the three TBR1 EvoTop stress tested isolates. We found that each TBR1 EvoTop isolate contained unique mutations in the “Antagonist of Mitotic exit Network” (*AMN1*) gene. The *AMN1* gene (Li et al., 2013; Yvert et al., 2003) is part of the *ACE2* regulon (Di Talia et al., 2009) that mediates the forward transition to multicellularity in yeast (Ratcliff, Fankhauser, Rogers, Greig, & Travisano, 2015). All isolates were monoclonal, except for the TBR1 A EvoTop isolate, for which sequencing indicated 2 clones. The *FLO11* gene of TBR1 encodes a surface flocculin that is required for biofilm cell‐surface adhesion and involved in cell‐cell adhesion (Lo & Dranginis, 1998; Reynolds & Fink, 2001). The TBR1 A EvoTop colony we isolated appears to have been founded by cells from 2 genetically distinct clones that may have adhered to each other. For that colony, there are two mutations in the* AMN1* gene that are within the 101 bp Illumina HiSeq reads. Each individual read that overlaps both locations contains either one mutation or the other.

The TBR1 A EvoTop isolate contained both a loss‐of‐start missense mutation (Met1Arg) and a stop‐gained nonsense mutation (Ser20*), which were present at approximately 72%, and 18% in the population, respectively. We did not observe any differences in the clump‐forming abilities over individual experiments that we started from single colonies of the TBR1 A EvoTop isolate, probably because both mutations should effectively knock out the gene, resulting in complete loss of Amn1p function and identical phenotypes. The TBR1 B EvoTop isolate contained a missense mutation in *AMN1* resulting from a Thr405Arg polymorphism. The TBR1 C EvoTop isolate also had a missense mutation in the *AMN1* gene, causing a Lys496Asn amino acid change. Histograms showing the distribution of clump diameters in SD‐tryp + 2% glucose medium of the TBR1 (isolates) and BY4742 ancestral and EvoTop variants indicate that the distribution of BY4742 clump diameters stayed consistent between the ancestral and EvoTop variants. However, the distribution of TBR1 EvoTop isolate clump diameters shifted away from the TBR1 ancestral distribution and closer to that of BY4742 (Figure 4a). The TBR1 B and C EvoTop isolates shared similar distributions of clumps to the TBR1 A EvoTop isolate (Figure 4b)**.** So, while the effects of the *AMN1* mutations in the TBR1 B EvoTop isolate (Thr405Arg) and the TBR1 C EvoTop isolate (Lys496Asn) are harder to predict, they likely resulted in decreased or lost function, given the dramatic shift in clump‐forming to “unicellular” phenotypes we observed (Figure 4).

Taken together, these findings suggest an essential role of the *AMN1* gene in the transition to unicellularity, which is consistent with the known role of Amn1p in regulating a cytokinesis gene network (Fang et al., 2018; Wang, Shirogane, Liu, Harper, & Elledge, 2003). Finally, to investigate the possible role of the *AMN1* sequence in the unicellularity of laboratory strains, we also compared the sequence of the TBR1 ancestor to the published sequence of the standard laboratory strain BY4742. This revealed an Asp368Val polymorphism that changes an acidic residue to a hydrophobic one and likely impairs the functionality of the Amn1p protein (Yvert et al., 2003).

### 3.4 Unicellularity weakens stress resistance, but might aid growth in normal conditions

**Figure 5:**
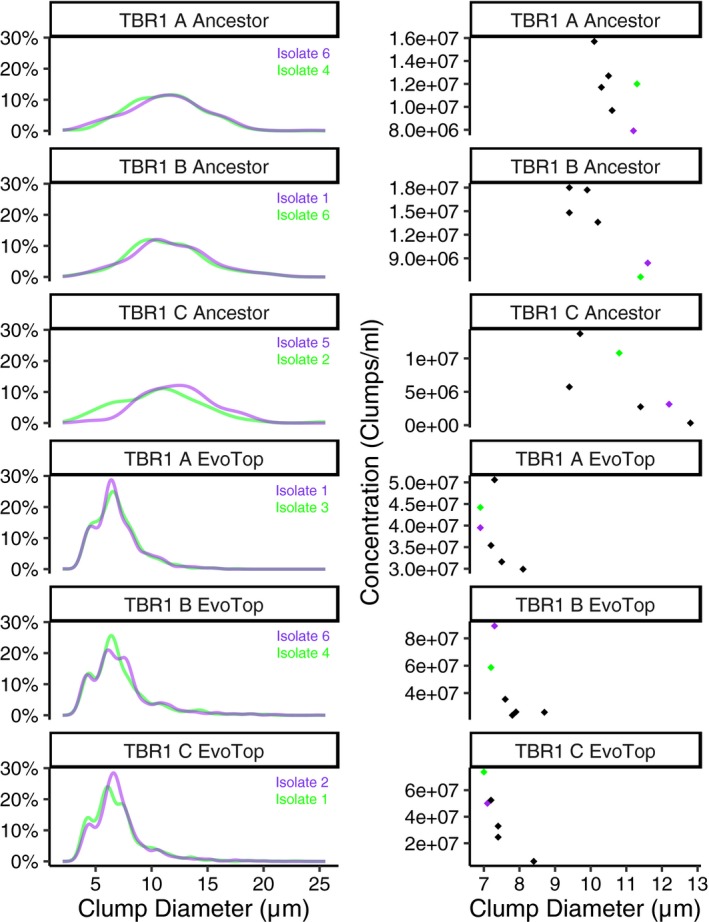
Clump diameter distribution (left) and average clump size by clump concentration (right) of ancestor and EvoTop isolates. Isolates shown in purple were used for the stress test experiments

**Figure 6:**
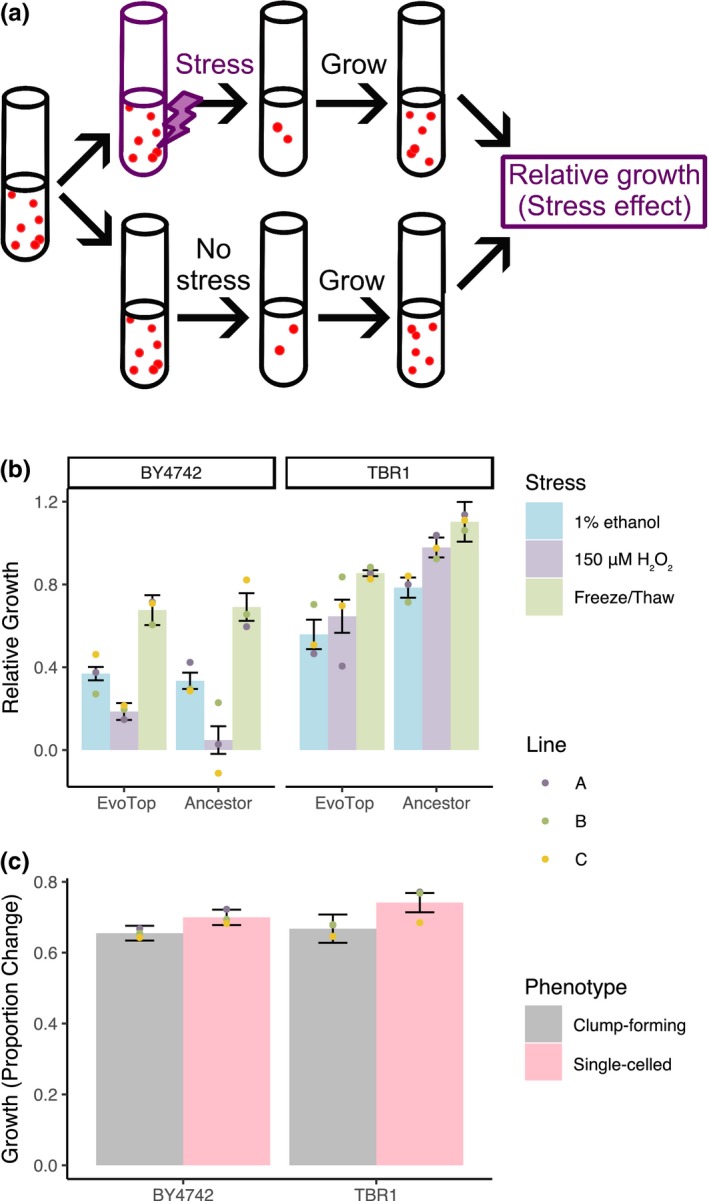
Clumping protects yeast from stress, but hinders growth in normal conditions. (a) Experimental procedure for testing the effects of three different forms of stress. Cells were diluted to equal concentrations, exposed to each stress, and then allowed to grow for 1.5 hr. The stress effect (relative growth) was the ratio of growth with stress to growth without stress. (b) Significant differences in the relative growth of stressed cells compared to unstressed controls indicated that single‐celled TBR1 EvoTops were less stress resistant than the ancestral clump‐forming TBR1 cells (three‐way variant (*F*(1, 36) = 25.16, *p* < 0.0001) by stress by line ANOVA; bars indicate mean relative growth, error bars indicate *SEM*, and dots indicate mean relative growth per line). In contrast, the relative growth of BY4742 ancestral and BY4742 EvoTop variants did not vary significantly from each other after stress treatment (*F*(1, 36) = 1.42, *p* = 0.241). (c) Under stress‐free conditions, clump‐forming cells had significantly lower growth over an hour and a half than single cells, but growth rates did not vary significantly from each other by strain or line (three‐way strain by phenotype (*F*(1, 96) = 4.09, *p* = 0.046) by line ANOVA; bars indicate mean growth, error bars indicate *SEM*, and dots indicate mean growth per line). The clump‐forming ancestral TBR1 cells had lower growth than EvoTop TBR1 single cells, while unstressed ancestral BY4742 single cells had higher growth than their clumpier EvoTop BY4742 counterparts

Next, we asked how unicellularity affects stress resistance compared to multicellularity. To compare the stress response of single‐celled and clump‐forming strains, we isolated colonies from each TBR1 EvoTop and ancestral line that typified their respective unicellular and clump‐forming phenotypes (see “TBR1 isolates” section and Figure 5). Then, we exposed each of these TBR1 isolates to three different stresses: rapid freeze/thaw, 150 μM hydrogen peroxide, and 1% exogenous ethanol, and measured their growth relative to unstressed cells over 1.5 hr. We also similarly compared the relative growth of BY4742 EvoTop and ancestral cells (Figure 6a).

A three‐way variant (TBR1 ancestor, TBR1 EvoTop) by stress treatment (freeze/thaw, 1% ethanol, 150 µM H_2_O_2_) by line (A, B, C) ANOVA revealed significant main effects of variant (*F*(1, 36) = 25.16, *p* < 0.0001) and stress treatment (*F*(2, 36) = 10.94, *p* = 0.0002) on relative growth of stressed cells, but no significant interactions (at a 0.05 alpha level). TBR1 EvoTop (single‐celled) lines grew significantly slower (*M* = 0.686, *SD* = 0.220) than the TBR1 ancestral clumping strains (*M* = 0.955, *SD* = 0.237) over 1.5 hr of exposure for all stresses. The TBR1 ancestor's relative growth rate was consistently higher than TBR1 EvoTop's growth rate after exposure to 1% ethanol (*M* = 0.785, *SD* = 0.146 vs. *M* = 0.559, *SD* = 0.213), 150 µM hydrogen peroxide (*M* = 0.979, *SD* = 0.144 vs. *M* = 0.646, *SD* = 0.241), and freeze/thaw treatment (*M* = 1.103, *SD* = 0.288 vs. *M* = 0.854, *SD* = 0.043). We examined contrasts of the estimated marginal means for variant conditioned by stress treatment and found that the TBR1 ancestral clump‐forming variant had significantly higher relative growth rates than the TBR1 EvoTop unicellular variant over all three stressors: 1% ethanol, *p* = 0.020; 150 µM hydrogen peroxide, *p* = 0.001; freeze/thaw, *p* = 0.011; Figure 6b. Therefore, the TBR1‐derived single‐celled EvoTop lines are less resistant to freeze/thaw, hydrogen peroxide, and ethanol stressors than the clumping TBR1 ancestral lines (Figure 6b).

There was not a significant main effect of replicate line (A, B, C) on relative growth of stressed cells (*F*(2, 36) = 0.59, *p* = 0.562). Freeze/thaw stress treatment cells (*M* = 0.979, *SD* = 0.237) had significantly higher relative growth rates than 1% ethanol (*M* = 0.672, *SD* = 0.212; Tukey post hoc test, *p* = 0.0001) or 150 µM H_2_O_2 _(*M* = 0.812, *SD* = 0.258; Tukey, *p* = 0.041) treated cells, which did not vary significantly from each other (Tukey, *p* = 0.095). We ruled out the potential growth effect of alcohol as a nutrient by growing the cells in media containing 2% glucose, which yeast cells strongly prefer for feeding compared to alcohol (which is the basis of winemaking). Overall, these results implied that clumping provides the benefit of protecting yeast cells from three different forms of environmental stress.

Next, we sought to separately determine if the experimental evolution procedure could somehow lower the stress response of EvoTop lines. To test this, we asked if the single‐celled BY4742 ancestral and EvoTop lines differed in their stress resistance. A three‐way variant (BY4742 ancestor, BY4742 EvoTop) by stress treatment (freeze/thaw, 1% ethanol, 150 µM H_2_O_2_) by line (A, B, C) ANOVA revealed a significant main effect of stress (*F*(2, 36) = 55.43, *p* < 0.0001), but no main effects of variant (*F*(1, 36) = 1.42, *p* = 0.241) or replicate line (*F*(2, 36) = 0.09, *p* = 0.918) on relative growth of stressed cells (Figure 6b). We found no significant interactions. BY4742 did have significantly higher growth rates after freeze/thaw treatment (*M* = 0.683, *SD* = 0.203) than 1% ethanol (*M* = 0.352, *SD* = 0.106; Tukey post hoc test, *p* < 0.0001) or 150 μM H_2_O_2_ (*M* = 0.117, *SD* = 0.176; Tukey, *p* < 0.0001) treatment, which were significantly different from each other (Tukey, *p* = 0.0003). However, BY4742 ancestral and EvoTop variants did not have significantly different growth rates after stress exposure. This indicates that BY4742 ancestral and EvoTop single cells and multiplets were similarly vulnerable to freeze/thaw, hydrogen peroxide, and ethanol stress (Figure 6b)**.** Consequently, the unicellular selection procedure, in and of itself, is unlikely to cause increased sensitivity in EvoTop lines.

Finally, to investigate whether the single‐celled phenotype may also have some benefits over multicellularity, we compared the growth of TBR1 and BY4742 single cells and clumps in the absence of stress. While the overall growth of TBR1 (*M* = 0.704, *SD* = 0.180) and BY4742 (*M* = 0.677, *SD* = 0.112) did not differ significantly from each other, a three‐way strain (*F*(1, 96) = 0.87, *p* = 0.354) by phenotype by line (*F*(4, 96) = 0.55, *p* = 0.696) ANOVA revealed a significant main effect of phenotype (*F*(1, 96) = 4.09, *p* = 0.046) on the growth rate of the unstressed cells (Figure 6c). There were no significant strain by phenotype (*F*(1, 96) = 0.25, *p* = 0.621) or phenotype by line (*F*(4, 96) = 0.10, *p* = 0.983) interactions. Single cells (*M* = 0.720, *SD* = 0.129) divided significantly faster than clump‐forming cells (*M* = 0.661, *SD* = 0.164). Accordingly, TBR1 EvoTop single cells (*M* = 0.741, *SD* = 0.142) grew faster than their clump‐forming TBR1 ancestral cells (*M* = 0.668, *SD* = 0.207) in normal conditions. Likewise, unstressed BY4742 ancestral single cells (*M* = 0.700, *SD* = 0.113) had slightly higher growth over an hour and a half than their unstressed clumpier EvoTop counterparts (*M* = 0.655, *SD* = 0.108; Figure 6c). Overall, we concluded that clumping hampered growth, implying that clumping multicellularity could be costly in the absence of stress. Implicitly, then unicellularity would be beneficial without stress. However, these potential benefits were insufficient to significantly reduce clump size for the TBR1 EvoControl variant relative to the ancestral variant after 4 weeks without counter‐gravitational selection (Figures 2 and 3b).

In summary, the multicellular clump‐forming yeast phenotype offers the benefit of increased stress resistance over single cells. Conversely, we found that clumping might be costly in normal conditions, arguing for some potential advantage of unicellularity over multicellularity in the absence of stress.

### 3.5 Other mutations in EvoTop isolates do not appear to affect stress resistance

In addition to the *AMN1* mutations we identified, TBR1 EvoTop isolate sequencing results revealed a few mutations that could potentially affect stress response (Table 1). The most notable of these is the Gln1442Lys missense mutation in the adenylate cyclase *CYR1* gene of the TBR1 B EvoTop isolate. *CYR1* is an essential gene that encodes adenylate cyclase, and null mutants are inviable (Giaever et al., 2002).

To help determine the impact of such mutations on stress resistance, we compared the stressed growth rates of EvoTop isolates from each line (A, B, C). The TBR1 B EvoTop isolate had a higher growth rate after stress exposure (*M* = 0.808, *SD* = 0.147) than the TBR1 A EvoTop isolate (*M* = 0.575, *SD* = 0.272) and the TBR1 C EvoTop isolate (*M* = 0.677, *SD* = 0.176; Figure 6b, TBR1 EvoTop lines). Following a three‐way variant (TBR1 ancestor, TBR1 EvoTop) by stress treatment (freeze/thaw, 1% ethanol, 150 µM H_2_O_2_) by line (A, B, C) ANOVA (see previous section), we examined contrasts of the estimated marginal means for line conditioned by variant. We found that the TBR1 B EvoTop isolate had a significantly higher relative growth rate after stress exposure than the TBR1 A EvoTop isolate (Tukey adjusted, *p* = 0.043). The TBR1 C EvoTop isolate did not vary significantly from the TBR1 A EvoTop isolate (Tukey adjusted, *p* = 0.520) or the TBR1 B EvoTop isolate (Tukey adjusted, *p* = 0.348; Figure 6b, TBR1 EvoTop lines). The TBR1 B EvoTop isolate was not only viable, but its relative growth after stress exposure was significantly higher than that of the TBR1 A EvoTop isolate, and it consistently had higher growth rates than the TBR1 A and C EvoTop isolates across all three stressors (Figure 6b, TBR1 EvoTop lines). This suggests that the *CYR1* mutation may not have had a negative effect on the TBR1 B EvoTop isolate's stress tolerance.

The TBR1 B EvoTop isolate also had a mutation in *OSW1*, and one intergenic to *YKE4* and *TIM44*. The TBR1 A EvoTop isolate contained mutations in *BAP2*, *DOT6*, and *VPS13*. While none of these mutations are in specific stress response genes, they may ultimately affect stress tolerance in these isolates. Interestingly, out of these five mutations, only the *DOT6* mutation in the TBR1 A EvoTop isolate was also called in the EvoTop pool it was isolated from. The other mutations were either present at too low a frequency to be identified during sequencing, or are truly novel to the isolates. The former is the likely case for the *OSW1* mutation in the TBR1 B EvoTop isolate, which can be found at low frequency (~3%) in the aligned reads from the TBR1 B EvoTop population. There is no evidence of the remaining three mutations in the files from their corresponding pools, but this alone is not enough evidence to determine whether the mutations are truly unique to the isolates or were simply at too low a frequency in the pools to show up under our given coverage. Aside from the *AMN1* mutation, we did not identify any other high frequency mutations in the TBR1 C EvoTop isolate. Notably, the relative stressed growth of the TBR1 C EvoTop isolate did not vary significantly from that of the TBR1 A or B EvoTop isolates (Figure 6b), and the overall relative stressed growth of the TBR1 EvoTop isolates was higher than that of the BY4742 ancestral and EvoTop variants (Figure 6b). This suggests that stress tolerance of the TBR1 EvoTop isolates has not been compromised by random mutations.

## 4 DISCUSSION

While the transition from unicellularity to multicellularity has been studied extensively, the reverse transition from multicellularity to unicellularity (Hammerschmid et al., 2014) has received less attention, especially without cheaters in eukaryotes. We obtained a reverse transition by EvoTop selection as a new exception from Dollo's Law (Dollo, 1893). We found that clumping protects from stresses but is costly in normal conditions. This may imply a trade‐off between growing fast and dying in stress (unicellular phenotypes) versus growing slower, but resisting stress (multicellular phenotypes). A similar trade‐off was recently observed in the evolution of engineered unicellular yeast cells under stress (Gonzalez et al., 2015). Such trade‐offs emerge from the pressure to satisfy two conflicting tasks, resolved by Pareto optimality in biological evolution (Shoval et al., 2012). Overall, these findings suggest that environmental stress could play a major role in the maintenance, or possibly even the emergence of multicellularity in other species, such as social amoebae (Gregor, Fujimoto, Masaki, & Sawai, 2010). These findings corroborate the protective role that clumping or other forms of multicellularity provide against predation (Brunke et al., 2014; Pentz et al., 2015) and environmental stress (Smukalla et al., 2008).

Both physical shielding and physiological changes appear to play a role in the increased stress tolerance of flocs compared to single cells (Smukalla et al., 2008). Along with being blocked from external stressors by exterior floc cells, internal floc cells have limited access to nutrients and oxygen. This leads to decreased growth as indicated by the downregulation of mitotic genes. There is a corresponding upregulation of genes associated with stress response (Smukalla et al., 2008). Smukalla and colleagues (2008) were able to test the effects of different stressors on flocculating and nonflocculating cells by breaking up flocs with EDTA after exposure to stress and measuring colony forming units. Our clumps formed from incomplete daughter cell separation do not lend themselves well to the colony forming unit assay, which led us to examine the effects of stress by calculating the relative growth of stressed cells compared to unstressed controls. Clumps are not easily broken up, and the mechanical method of sonication we used could lyse and kill the cells. While these cells are still countable in our initial and final time points, they would not show up in a colony forming unit assay. Similarly, very small clumps with countable cells existed in the TBR1 samples after sonication, but these multiple cells would have only formed a single colony. We sonicated samples of the TBR1 cultures that we took to count, but the cells we measured for growth were not exposed to sonication until we were ready to obtain single‐cell counts after they had undergone 1.5 hr of growth.

We found that haploid yeast clumps are more resistant than single cells against both physical (freeze/thaw) and chemical (ethanol and hydrogen peroxide) stressors. Interestingly, protection from freeze/thaw appears to be a benefit that the flocculating multicellular form does not possess (Smukalla et al., 2008). Strikingly, not only did the TBR1 ancestral cells tolerate freeze/thaw treatment, they grew ~110% better than their corresponding untreated controls. An explanation is that freeze/thaw cycles impose mechanical stress (Harju, Fedosyuk, & Peterson, 2004), which could cause clump splitting, improving access to nutrients. Interestingly, the unicellular BY4742 strain also started forming more multiplets during evolution, which may have been selected for because multiplets mitigate bud scars' vulnerability to mechanical stress from vortexing (Chaudhari et al., 2012). The higher stress tolerance of clumps may be due to either physical shielding of interior cells from external stressors, replicative aging, physiological changes, or a combination of such factors (Brachmann et al., 1998; Smukalla et al., 2008).

Our findings suggest that clumping may create phenotype switching and deterministic heterogeneity, which plays a role in the drug resistance of microbial pathogens (Aldridge et al., 2012). Indeed, some *S. cerevisiae* strains emerging as opportunistic pathogens (Wei et al., 2007) contain the same *AMN1* allele as the clump‐forming TBR1 ancestor. Moreover, long‐term evolution of *Candida glabrata* with macrophages causes the evolution of a filamentous multicellular form due to incomplete daughter cell separation (Brunke et al., 2014). Further work is needed to examine what role clump formation might play in yeast infections, but one can envision clumps forming stress resistant propagules. Future studies of clumping and other forms of multicellularity in infectious yeasts and other microbes should lead to improved antibiotic efficiency, addressing the emerging global threat of drug resistant infections.

## CONFLICT OF INTEREST

None declared.

## AUTHOR CONTRIBUTIONS

J.K.F. and G.B. designed the research; L.C. performed fluorescent labeling and imaging of yeast cells, J.K.F. performed the majority of experiments, data analysis, and statistics; J.K.F. and G.B. wrote the paper.

## Data Availability

Whole‐genome sequence data are available from NCBI assembled at BioProject PRJNA388338. Additional data are available at https://openwetware.org/wiki/File:Kuzdzal-Fick_et_al_2019.zip.
